# From Prevalence to Incidence: Validation of Healthcare-Associated Infection Incidence Estimations from Prevalence Data in a Serbian University Hospital

**DOI:** 10.3390/jcm15207981

**Published:** 2026-10-15

**Authors:** Nikola Milenkovic, Ljiljana Markovic-Denic, Sonja Novak, Bojan Stanojevic, Dane Krtinić, Gorana Nedin Rankovic, Aleksandar Nikolic, Vladimir Nikolić, Aleksandar Rankovic

**Affiliations:** 1 Department of Infectious Diseases and Epidemiology, Faculty of Medicine, University of Niš, 18000 Niš, Serbia; nikola.milenkovic@medfak.ni.ac.rs (N.M.); sonja.novak@medfak.ni.ac.rs (S.N.); aleksandar.rankovic@medfak.ni.ac.rs (A.R.); 2 Department of Sanitary and Epidemiological Surveillance, University Clinical Center Niš, 18000 Niš, Serbia; 3 Institute of Epidemiology, Faculty of Medicine, University of Belgrade, 11000 Belgrade, Serbia; vladimir.nikolic@med.bg.ac.rs; 4 Department of Healthcare-Associated Infections Prevention, University Hospital Medical Center “Bezanijska Kosa”, 11070 Belgrade, Serbia; 5 Department of Pharmacology with Toxicology, Faculty of Medicine, University of Niš, 18000 Niš, Serbia; dane.krtinic@medfak.ni.ac.rs (D.K.); gorana.nedin.rankovic@medfak.ni.ac.rs (G.N.R.); 6 Clinic of Oncology, University Clinical Center Niš, 18000 Niš, Serbia; 7 Department of Surgery and Anesthesiology with Reanimatology, Faculty of Medicine, University of Niš, 18000 Niš, Serbia; aleksandar.nikolic@medfak.ni.ac.rs; 8 Clinic for Anesthesia and Intensive Care, University Clinical Center Niš, 18000 Niš, Serbia; 9 Clinic for Infectious Diseases, University Clinical Center Niš, 18000 Niš, Serbia

**Keywords:** healthcare-associated infections, point prevalence survey, cumulative incidence, continuous surveillance, Rhame and Sudderth formula

## Abstract

**Introduction:** Continuous prospective surveillance is considered the reference method for monitoring healthcare-associated infections (HAIs), but it requires substantial personnel and organizational resources. Point prevalence surveys provide a simpler alternative, although they primarily measure prevalence rather than incidence. The aim of this study was to evaluate the validity of the estimation of cumulative incidence of healthcare-associated infections (HAIs) from a point prevalence survey (PPS) across different hospital wards. **Methods:** A HAI PPS was conducted between 14 November and 2 December 2022, at the University Clinical Center Nis, Serbia, using the European Centre for Disease Prevention and Control (ECDC) protocol. Data from 774 patients were analyzed. Prevalence data from the Surgical Intensive Care Unit (SICU) and the Orthopedics and Traumatology department were converted into estimated cumulative incidence using the Rhame and Sudderth formula. These estimates were compared against the actual 2022 cumulative incidence obtained via continuous surveillance. **Results:** The overall HAI prevalence was 3.6% (28 HAIs among 27 patients; 95% CI: 2.5–5.2%). In the SICU, the formula-derived calculated incidence (5.3 per 100 admissions) demonstrated an excellent fit with the observed continuous surveillance incidence (5.54 per 100 admissions; ratio: 0.96), falling well within its 95% CI (4.24–7.22%). Conversely, in the Orthopedics department, the formula significantly overestimated the true incidence by a factor of 10.7 (2.6 vs. 0.24 per 100 admissions), lying far above the upper limit of the observed 95% CI (0.09–0.62%). **Conclusions:** Converting prevalence data into cumulative incidence estimates is an accurate tool for estimating HAI incidence in stable, high-risk environments like intensive care units. However, it should be applied with extreme caution in low-prevalence specialty wards, where small sample sizes and individual outliers can lead to profound misestimation.

## 1. Introduction

Healthcare-associated infections (HAIs) represent a major public health and patient-safety challenge worldwide, contributing substantially to patient morbidity, prolonged hospital stays, excess mortality, antimicrobial resistance, and healthcare costs [1]. Their burden varies considerably across countries, healthcare systems, clinical settings, and patient populations. A global systematic review and meta-analysis demonstrated substantial geographical and ward-level variation in HAI prevalence, with particularly high rates reported in intensive care units, neonatal units, and transplant settings [2]. More recent evidence confirms the particularly high burden among critically ill patients. A systematic review and meta-analysis of 21 studies including 15,966 adult intensive care unit (ICU) patients found that pneumonia, bloodstream infections, and urinary tract infections were among the most frequently reported HAIs, while invasive procedures, antimicrobial exposure, and underlying health conditions were important risk factors [3].

The contemporary epidemiology of HAIs is further complicated by the increasing burden of antimicrobial resistance and the important role of invasive devices and procedures. Recent evidence highlights device-associated infections, including catheter-associated urinary tract infections, central line-associated bloodstream infections, and ventilator-associated pneumonia, as major components of HAI burden, frequently involving multidrug-resistant pathogens [4]. Regional studies also demonstrate marked variability in infection frequency and microbiological patterns. For example, a recent multicenter study from Lebanon reported surgical site infections in 0.91% of surgical patients, with Gram-negative bacteria predominating and substantial antimicrobial resistance among the isolated organisms [5]. These variations underscore the importance of robust and standardized surveillance systems for accurately describing and comparing the burden of HAIs across different clinical settings.

Accurate monitoring of HAIs is essential for evaluating the quality of patient care and measuring the effectiveness of infection prevention and control (IPC) programs [6]. Continuous prospective surveillance is considered the reference approach for epidemiological monitoring of HAIs [7]. However, maintaining comprehensive continuous surveillance across all hospital departments requires substantial human resources, continuous training, and robust information technology infrastructure—resources that may be limited or unevenly distributed across healthcare facilities. Point prevalence studies provide a simpler, rapid, and relatively inexpensive approach for assessing the epidemiological situation regarding HAIs [8]. While a point prevalence survey (PPS) provides a practical “snapshot” of infection burden, it primarily measures prevalence rather than cumulative incidence, which is generally more informative for assessing infection risk and monitoring changes over time.

The point prevalence survey (PPS) method was standardized for EU countries in 2010 [9] and has since been used with minor variations across three consecutive PPS cycles in European hospitals. Serbia participated in the last two EU HAI PPS, in 2017 and 2022 [10]. Moreover, the implementation of the HAI PPS at the national level every five years is mandated by national legal regulations [11].

To maximize the utility of prevalence data, mathematical models have been developed to convert point prevalence indicators into estimated cumulative incidence rates. The most prominent of these is the estimation method developed by Rhame and Sudderth, which relies on the point prevalence, the average length of stay for all patients, and the duration of the infection [12]. Although this approach has been used for years [13], the real-world applicability of this formula remains a subject of ongoing validation, particularly when applied across diverse clinical environments ranging from high-risk intensive care units to specialized surgical and medical wards [14].

This comparative study aims to evaluate the validity and accuracy of the Rhame and Sudderth formula by converting HAI prevalence data obtained from a tertiary hospital using a standardized European Centre for Disease Prevention and Control (ECDC)-based methodology [10,15] into cumulative incidence estimates and to compare these calculated estimates with actual, year-long cumulative incidence rates obtained through continuous prospective surveillance within the same wards. Subsequently, these calculated metrics will be compared with actual, year-long cumulative incidence rates obtained through continuous prospective surveillance within the same wards.

## 2. Materials and Methods

### 2.1. Prevalence Survey

The HAI PPS was conducted at the University Clinical Center Nis (UKC Nis) between 14 November and 2 December 2022. This tertiary care hospital has a capacity of 1954 beds and 502 hospital rooms, of which 22 are single beds. Approximately 50,000 patients are treated annually at the facility, where around 13,000 operations, 3000 deliveries, and 10,000 dialysis procedures are performed. The HAI PPS was conducted as part of the national prevalence study in Serbia, aligned with the implementation timeframe of other European Union (EU) countries. The methods used for this survey followed the detailed protocol of the ECDC [15], which was translated into the Serbian language [10]. Briefly, patients who were hospitalized at 8:00 a.m. on the day of the survey in a given department and those who had not been discharged at the time of the study were included. Conversely, patients undergoing day therapy or day surgery, patients examined exclusively in outpatient clinics, patients in the emergency room, and outpatients on a chronic dialysis program were excluded.

Although hospital-wide data collection was conducted between 14 November and 2 December 2022, each ward was surveyed cross-sectionally on its designated survey day in accordance with the ECDC PPS methodology; the prevalence estimates therefore represent point prevalence rather than prevalence accumulated over this period.

No age restrictions were applied. All patients meeting the ECDC PPS eligibility criteria were included irrespective of age. Furthermore, all active types and sites of healthcare-associated infections (HAIs) were considered for inclusion, provided they met the standard clinical and laboratory diagnostic criteria defined by the ECDC protocol [15].

### 2.2. Data Collection

Data were collected by a single, dedicated team from the hospital’s infection prevention and control (IPC) department, consisting of IPC nurses and IPC physicians–epidemiologists. Data collection utilized a standardized paper questionnaire, which had been validated for clarity and comprehensibility at the national level by the HAI PPS study team. Anonymized data from the paper questionnaires were entered into a predefined database in the Helics.win software [16] and a standardized hospital Excel spreadsheet (using Microsoft Excel 2021 (Microsoft Corporation, Redmond, WA, USA)), with prior cross-consistency checks performed between both databases. Data from Helics.win were subsequently submitted to the ECDC.

Data on the hospital- and department-level characteristics were collected in a separate questionnaire, which included hospital type, specialty, size, total annual patient admissions, and the number of eligible patients present in the ward at the designated morning hour. Patient-level data included age, gender, admission date, and presence of invasive devices (central vascular catheters, urinary catheters, or mechanical ventilation). These parameters, alongside the duration of hospitalization prior to the survey, were recorded to evaluate the exposure to potential causes and risk factors for HAIs. For patients presenting with an active infection during the PPS, additional data were gathered regarding specific criteria-matching infection sites (e.g., respiratory, urinary tract, surgical site, or bloodstream infections), onset date, and documented associated microbial pathogens, which allowed for the analysis of the microbiological etiology behind the infections.

### 2.3. Definition of Healthcare-Associated Infection

To identify the presence of an HAI, the standard definitions of the ECDC were applied [15], utilizing the official Serbian translation [17]. All active HAIs meeting the ECDC case definitions applicable to the PPS protocol were eligible for inclusion, irrespective of infection site.

### 2.4. Incidence Data

The hospital IPC team’s continuous surveillance database served as the primary data source for calculating the actual cumulative incidence for the year 2022. The hospital information system was utilized to extract precise lengths of hospitalization for the monitored patient cohort.

### 2.5. Conversion of Prevalence Data to Incidence Estimates

HAI prevalence was converted into estimated cumulative incidence using the Rhame and Sudderth formula: I = P [LA/(LN − INT)]. Here, I is cumulative incidence, P is prevalence, LA is the average length of hospitalization of all the hospitalized patients during the study period, LN is the average length of hospitalization of patients having one or more HAIs, and INT is the average interval between admission and onset of the first HAI for patients having one or more HAIs [12]. We included only the Orthopedic Surgery department and SICU in the analysis, where active continuous surveillance of HAIs was conducted in 2022, as this was necessary to compare estimated incidence and density incidence. Other hospital sectors, including the heterogeneous Internal Medicine department (which comprises 12 distinct sub-specialty clinics), do not undergo continuous annual surveillance, thereby precluding them from this validation study.

### 2.6. Statistical Analysis

The HAI point prevalence was calculated to express the percentage of infected patients on the specific survey date within each department. The numerator was defined either as a patient with at least one HAI (for calculating the prevalence of patients with HAIs) or as the total number of reported HAIs on the survey date (for calculating the overall prevalence of HAIs). The denominator consisted of all eligible patients hospitalized in the respective ward on the day of the survey. The observed cumulative HAI incidence was calculated as the number of new HAIs developed during the continuous surveillance period of 2022 divided by the total number of patients initially “at risk” who entered that period free of infection. Both prevalence and cumulative incidence are expressed as percentages, as both indicators represent mathematical proportions. For descriptive presentation, age was categorized according to the predefined age-group structure used in the national HAI PPS database: 0–14, 15–43, 44–64, and ≥65 years. Statistical analyses were performed using SPSS version 26.0 (IBM Corp., Armonk, NY, USA).

## 3. Results

### 3.1. Study Population in HAI PPS

A total of 774 patients were included in the PPS; of those, 401 (51.8%) were male, and 373 (48.2%) were female. The mean age was 49.3 ± 24.6 years (median 54 years, range 0.08–96 years); patients aged 65 years and over formed the largest age group (281 patients, 36.3%), while children and adolescents aged 0–14 years were the smallest (97 patients, 12.5%). Underlying disease was classified as non-fatal in 597 patients (77.1%), and 214 patients (27.6%) had undergone surgery during the current admission. On the day of the survey, 177 patients (22.9%) had a urinary catheter, 38 (4.9%) had a central vascular catheter, and 25 (3.2%) were intubated. The characteristics of the surveyed population are summarized in Table 1.

Among the surveyed patients, 27 patients had a total of 28 active healthcare-associated infections (HAIs), resulting in an overall HAI prevalence of 3.6% (95% CI: 2.5–5.2%). One patient had two concurrent HAIs at the time of the survey. The distribution of HAIs across hospital departments is presented in Table 2.

The highest prevalence was observed in the Surgical Intensive Care Unit (SICU) (11.8%). Among the departments with registered infections, the lowest prevalence was found in the Orthopedic and Traumatology department (2.5%), while several departments (General Surgery, Gynecology/Obstetrics, Psychiatry, Rehabilitation, and Other ICU) recorded no active infections during the survey.

### 3.2. Sites of Infection

The distribution of the 28 infections by site is shown in Table 3. Hospital-acquired COVID-19 was the single most frequent condition (13 infections, 46.4%), followed by urinary tract infections (4, 14.3%) and surgical site infections (3, 10.7%; two superficial and one deep incisional). Bloodstream infections and bronchitis accounted for two infections each (7.1%), and one infection each was recorded for pneumonia, skin and soft-tissue infection (pressure ulcer), infection of the central nervous system, and neonatal sepsis (3.6% each). The prevalence of surgical site infection among the patients operated on during the current admission was 1.4 per 100 operated patients.

### 3.3. Causative Microorganisms

A total of 31 microorganisms were isolated from the 28 infections (Table 4). SARS-CoV-2 was the most frequently identified agent (13 isolates, 41.9%). Among the bacterial isolates, *Klebsiella pneumoniae* was the most common (4, 12.9%), followed by *Pseudomonas aeruginosa* (3, 9.7%), *Acinetobacter baumannii* and *Escherichia coli* (2 each, 6.5%), and one isolate each of *Enterococcus faecalis*, *Serratia marcescens,* and *Staphylococcus aureus* (3.2% each); the remaining four isolates (12.9%) belonged to other species.

### 3.4. Incidence Estimation from Prevalence

This analysis was conducted based on data from the Orthopedics and SICU departments. To account for the potential confounding effects of the pandemic, a sensitivity analysis focusing on healthcare-associated COVID-19 cases was performed. However, zero cases of healthcare-associated COVID-19 were identified in either the SICU or the Orthopedics and Traumatology department during the PPS timeframe, confirming that the primary comparative results remained unaffected by COVID-19 infections.

In the SICU, the calculated cumulative incidence using the Rhame and Sudderth formula was 5.3 infections per 100 admissions (LA = 5.7 days, LN = 22.0 days, INT = 9.3 days). Continuous surveillance in the same ward during 2022 recorded 51 HAIs among 920 discharges, yielding an observed cumulative incidence of 5.54 per 100 admissions (95% CI: 4.24–7.22%). The calculated value underestimated the observed value by only 4% (ratio 0.96) and fell within the 95% confidence interval of the continuous surveillance data (Table 5).

Conversely, in the Orthopedics and Traumatology department, the formula yielded a calculated cumulative incidence of 2.6 infections per 100 admissions (LA = 7.0 days, LN = 8.8 days, INT = 2.0 days). Continuous surveillance recorded only 4 HAIs among 1668 discharges, resulting in an observed cumulative incidence of 0.24 per 100 admissions (95% CI: 0.09–0.62%). Consequently, the prevalence-derived calculated incidence exceeded the observed continuous surveillance incidence by a factor of 10.7, placing it significantly above the upper limit of its 95% confidence interval (Table 5).

## 4. Discussion

This study evaluated the validity of estimating the cumulative incidence of HAIs from PPS data using the Rhame and Sudderth formula across distinct hospital settings. Because of continuous HAI surveillance in the hospital and the cumulative data collected over the same period across the same departments, it was possible to evaluate the reliability of the prevalence-to-incidence conversion in two units. Our findings demonstrate that while the formula provides an exceptionally accurate estimation in highly stable, high-risk environments like the Surgical Intensive Care Unit (SICU), it significantly overestimates incidence in specialty wards with lower baseline infection rates and shorter post-infection stays, such as the Orthopedics and Traumatology department. 

The overall HAI prevalence of 3.6% observed in our PPS at the University Clinical Center Niš is lower than the European average of 6.5% reported in the ECDC wide-ranging PPS [18]. Moreover, this prevalence is lower than the average prevalence in all acute care hospitals in Serbia (5.3%) [10] and lower than that obtained from another University Clinical Center (8.6%) [19]. However, this overall figure must be interpreted with caution due to the highly uneven distribution of infections across departments. The heavy concentration of risk was distinctly visible in the SICU, which exhibited the highest prevalence (11.8%). This is consistent with the clinical profile of the unit, characterized by high device utilization rates—including mechanical ventilation, central venous catheters, and urinary catheters—which are well-documented independent risk factors for severe HAIs.

The core methodological finding of this study lies in the contrasting performance of the Rhame and Sudderth formulas between the two analyzed wards. 

In the SICU, the calculated prevalence-derived incidence (5.3 per 100 admissions) showed similar values to the observed cumulative incidence recorded during annual surveillance (5.54 per 100 admissions), with remarkable precision, underestimating it by a mere 4% (ratio 0.96). This exceptional fit can be attributed to the SICU closely satisfying the steady-state assumptions required by the Rhame and Sudderth model. In such units, admission rates, average lengths of stay (LA), and the dynamics of infection development remain relatively uniform over time. Furthermore, the substantial duration of infections in the SICU (estimated at 12.7 days) ensures that the single-day “snapshot” of a PPS captures a representative cross-section of the ongoing disease burden. However, there are also opposite results from other studies. In a five-year study conducted in two intensive care units in France [20], the calculated prevalence-derived incidence was 24.5%, whereas the actual recorded incidence was 51.4%; thus, the estimated rate was almost twice as low as the rate obtained through incidence surveillance. Indirect estimation of incidence in intensive care units has substantial methodological limitations. Prolonged hospital stays, complex clinical courses, and the later onset of infections in critically ill patients may violate the assumptions underlying this model, as previously described in other studies [21]. An additional limitation is the assumption that multiple infections occurring in the same patient are independent. In critically ill patients, however, successive HAIs are often related through common underlying risk factors, including disease severity, prolonged hospitalization, and cumulative exposure to invasive devices; therefore, this assumption may not hold in ICU populations [12]. For example, Haore et al. [22], who applied prevalence-to-incidence conversion of bloodstream HAIs, concluded that this approach was not valid since the chance of a bloodstream infection developing was linked to the patient’s existing health issues, not to their extended time in the hospital. These methodological and biological discrepancies emphasize that mathematical accuracy cannot fully substitute for clinical plausibility, particularly when standardized formulas are applied to highly complex, heterogeneous patient populations like those in the ICU.

In sharp contrast, the formula failed to provide a reliable estimate for the Orthopedics and Traumatology department, overestimating the true incidence by a factor of 10.7 (2.6 vs. 0.24 per 100 admissions). This massive discrepancy highlights a well-known mathematical vulnerability of the Rhame and Sudderth formula when applied to low-prevalence settings with small sample sizes. In our case, the orthopedics ward yielded only a single active infection on the survey day among 40 surveyed patients. Because only a single patient with an active HAI was identified during the PPS, the values of LN and INT were effectively determined by that individual case, making the resulting conversion factor highly sensitive to patient-level variation (LA/LN-INT). Consequently, the formula projects this single, unrepresentative case onto the entire annual denominator, leading to an exaggerated cumulative incidence estimate that lies far outside the 95% confidence interval of the actual surveillance data. Moreover, this discrepancy may indicate possible underreporting of HAIs during routine surveillance in this hospital department, particularly surgical site infections (SSIs), which frequently manifest after patients have been discharged from the hospital. Findings from two Dutch surveys across 58 hospitals demonstrated that prevalence surveys cannot substitute for incidence surveillance of SSIs. Due to their cross-sectional nature, these surveys are inherently prone to length-time bias—oversampling prolonged infections while failing to capture a substantial proportion of post-discharge SSIs [23,24]. 

Another critical factor that shaped our results was the high prevalence of hospital-acquired COVID-19, which constituted nearly half (46.4%) of all detected HAIs, with SARS-CoV-2 being the most frequently isolated pathogen (41.9%). Conducted in late 2022, our study captured a period when hospital-acquired transmission of COVID-19 was still present in tertiary care facilities. The inclusion of an acute, highly transmissible respiratory pathogen introduces a significant distortion into the steady-state assumption of the Rhame and Sudderth formula. Unlike traditional bacterial HAIs (e.g., *Klebsiella pneumoniae* or *Pseudomonas aeruginosa*), which are heavily linked to invasive devices and prolonged hospitalization, hospital-acquired COVID-19 may occur in temporally clustered transmission events. Previous studies have also shown that healthcare-associated SARS-CoV-2 infections vary substantially across epidemic periods and that attribution to healthcare acquisition can be complicated by variable incubation periods and asymptomatic transmission [25]. This clustering may temporarily skew point prevalence upward, causing any back-calculated annual incidence to be overrepresented unless continuous surveillance adequately captures temporal variation in transmission.

Effective HAI prevention depends not only on surveillance but also on healthcare workers’ adherence to infection prevention and control practices. Recent KAP studies have shown that good knowledge does not always translate into adequate practice, highlighting the value of regular assessment of healthcare workers’ knowledge, attitudes, and practices to identify gaps and guide targeted training and reinforcement of standard precautions [26,27].

Antimicrobial resistance further increases the clinical burden of HAIs by limiting treatment options and complicating infection control. Recent molecular epidemiological studies of surgical site infections have demonstrated a high prevalence of multidrug-resistant organisms and clinically important resistance determinants, including *mecA*, *bla*CTX-M, and *bla*NDM, supporting the need for continuous microbiological surveillance, antimicrobial stewardship, and strict infection prevention measures [28].

In addition to antimicrobial stewardship and surveillance, several innovative approaches are being explored to address antimicrobial resistance. These include bacteriophage therapy, which offers pathogen-specific antibacterial activity; siderophore-based “Trojan Horse” antibiotics that exploit bacterial iron-uptake systems to facilitate drug entry into Gram-negative bacteria; and strategies targeting bacterial metallophore synthesis or transport, which may reduce virulence and provide new therapeutic targets [29,30,31].

The primary strength of this study is the direct, parallel comparison of prevalence-derived estimates against a robust, year-long continuous surveillance gold standard in the same hospital wards. This design allowed us to pinpoint exactly where the mathematical assumptions of epidemiologic models clash with clinical reality. However, some limitations must be acknowledged. First, the sample sizes for individual ward sub-analyses during the PPS (e.g., n = 17 in SICU and n = 40 in Orthopedics) were relatively small, which amplifies the statistical weight of individual outliers. In such limited cohorts, a single patient with an HAI represents a disproportionately large percentage of the sample. When converting these data using mathematical models, even just one or two outliers can significantly distort the projected cumulative incidence. Second, the single-day nature of the PPS means seasonal variations in hospital admissions or localized outbreaks (such as the observed COVID-19 cases) could not be smoothed out, unlike in the continuous surveillance database. Because a PPS provides only a static cross-sectional snapshot, it is structurally susceptible to capturing these non-representative epidemiological spikes. This has profound implications for data interpretation: if a survey happens to coincide with a temporary cluster of infections, the mathematical formula will artificially project this spike across the entire year, leading to a severe overestimation of the annual infection burden. Consequently, while the Rhame–Sudderth formula remains a valuable tool for stable, high-risk environments, its results in low-prevalence specialty wards, such as in our Orthopedics department, must be interpreted with extreme caution, as they are highly sensitive to temporary, non-representative fluctuations. The main limitation of this study is the small number of HAIs at the time of PPS conducted. With a greater number of HAIs, it would be possible to calculate the cumulative incidence, based on prevalence, for different HAI types, as demonstrated in one national study [32]. By comparing the incidences obtained from continuous surveillance and the prevalence-derived incidence for HAIs such as bloodstream infections, urinary tract infections, pneumonia, etc., it would be possible to see for which types of HAIs the mathematical formula can be most accurately applied. Another limitation is that the study was conducted in a single tertiary care hospital, which may limit the generalizability of the findings to other institutions with different patient populations, surveillance practices, and organizational structures. Multicenter validation would therefore be valuable to assess the reproducibility of prevalence-to-incidence conversion across different healthcare settings. Although the data were collected in 2022, the primary objective of this study was methodological rather than descriptive: to evaluate the agreement between prevalence-derived incidence estimates and continuous surveillance data obtained during the same period. Therefore, the validity of the comparison is not dependent on the data representing the current epidemiological situation. Nevertheless, changes in HAI epidemiology, circulating pathogens, and healthcare practices since 2022 should be considered when interpreting the descriptive prevalence findings.

Despite these limitations, combining data from prevalence studies with data from continuous surveillance provides a more comprehensive assessment of the burden of HAIs, particularly with regard to differences between individual clinical departments and organizational units.

## 5. Conclusions

In conclusion, the Rhame and Sudderth formula remains a highly valuable, resource-efficient tool for estimating HAI incidence, but its application must be strictly context-dependent. It can safely replace costly continuous surveillance in intensive care units and high-turnover, high-risk wards where steady-state dynamics apply. Conversely, for standard specialty wards with lower infection rates, incidence estimates derived from a single-day PPS should be treated with extreme caution, as small fluctuations in point prevalence can lead to profound overestimations of the annual infection burden. For these low-risk settings, targeted continuous surveillance remains indispensable.

## Figures and Tables

**Table 1:** Characteristics of the 774 patients included in the point prevalence survey, University Clinical Centre Niš, November–December 2022.

Characteristic	*n*	%
Sex		
Male	401	51.8
Female	373	48.2
Age group (years)		
0–14	97	12.5
15–43	179	23.1
44–64	217	28.0
≥65	281	36.3
McCabe score		
Non-fatal disease (>5 years)	597	77.1
Ultimately fatal disease (1–5 years)	140	18.1
Rapidly fatal disease (<1 year)	20	2.6
Unknown	17	2.2
Surgery during current admission	214	27.6
Invasive devices in situ		
Urinary catheter	177	22.9
Central vascular catheter	38	4.9
Intubation	25	3.2

**Table 2:** Prevalence of HAIs in different hospital departments.

Department	No of Patients	No of HAIs	HAI Prevalence (%)	95%CI * (%)
General surgery	179	0	0.0	0.0–2.1
Orthopedic and traumatology	40	1	2.5	0.4–12.9
Surgical intensive care unit	17	2	11.8	3.3–34.3
Internal medicine	300	20	6.7	4.4–10.1
Other intensive care unit	18	0	0.0	0.0–17.6
Gynecology/obstetrics	94	0	0.0	0.0–3.9
Psychiatry	46	0	0.0	0.0–7.7
Pediatrics	41	3	7.3	2.5–19.4
Rehabilitation	39	0	0.0	0.0–8.6
Total	774	28	3.6	2.5–5.2

* Wilson 95% CI.

**Table 3:** Healthcare-associated infections by site (n = 28).

Site of Infection	*n*	%
COVID-19 (multisystem)	13	46.4
Urinary tract infection	4	14.3
Surgical site infection	3	10.7
Superficial incisional	2	7.1
Deep incisional	1	3.6
Bloodstream infection	2	7.1
Bronchitis	2	7.1
Pneumonia	1	3.6
Skin and soft-tissue infection (pressure ulcer)	1	3.6
Central nervous system infection	1	3.6
Neonatal sepsis	1	3.6
Total	28	100.0

Other microorganisms included *Klebsiella* spp., *K. pneumoniae*, and other *Acinetobacter* spp.

**Table 4:** Microorganisms isolated from healthcare-associated infections.

Microorganism	*n*	%
SARS-CoV-2	13	41.9
*Klebsiella pneumoniae*	4	12.9
*Pseudomonas aeruginosa*	3	9.7
*Acinetobacter baumannii*	2	6.5
*Escherichia coli*	2	6.5
*Enterococcus faecalis*	1	3.2
*Serratia marcescens*	1	3.2
*Staphylococcus aureus*	1	3.2
Other	4	12.9
Total	31	100.0

**Table 5:** Calculated incidence and cumulative incidence obtained through continuous surveillance in intensive care and orthopedic/traumatology wards.

Parameter	Surgical Intensive Care Unit	Orthopedics and Traumatology
Patients surveyed, *n*	17	40
Patients with HAIs, *n*	2	1
Prevalence (P), %	11.8	2.5
Mean length of stay, all patients (LA), days	5.7	7.0
Mean length of stay, patients with HAIs (LN), days	22.0	8.8
Mean interval from admission to onset (INT), days	9.3	2.0
Estimated duration of infection (LN − INT), days	12.7	6.8
Conversion factor LA/(LN − INT)	0.45	1.03
Calculated cumulative incidence (I), per 100 admissions	5.3	2.6
Discharges in 2022, *n*	920	1668
HAIs recorded by continuous surveillance in 2022, *n*	51	4
Observed cumulative incidence, per 100 admissions (95% CI)	5.54 (4.24–7.22)	0.24 (0.09–0.62)
Ratio of calculated–observed	0.96	10.7
Prevalence back-calculated from observed incidence, %	12.4	0.2

## Data Availability

The data presented in this study are available upon request from the corresponding author due to legal reasons.

## Abbreviations

The following abbreviations are used in this manuscript:

HAIs	Healthcare-associated infections
PPS	Point prevalence survey
ECDC	European Centre for Disease Prevention and Control
SICU	Surgical Intensive Care Unit
IPC	Infection prevention and control
UKC	University Clinical Center
P	Prevalence
I	Incidence
LA	Average length of hospitalization of all hospitalized patients
LN	Average length of hospitalization of patients having HAIs
INT	Average interval between admission and onset of the first HAI
